# Winter Course of Lectures

**Published:** 1859-10

**Authors:** 


WINTER COURSE OF LECTURES.

It will be seen from the advertisement on the cover of our journal, that the Preparatory Course in the Atlanta Medical College, will commence on Monday, the 7th Nov., and continue four months. We were fully impressed during the past winter with the importance of this Course, feeling confident, that by this addition to our regular Course, we offer greatly increased facilities to the student. By this arrangement, it will be seen, that we teach *eight*

months instead of *four*, without extra charge, making *sixteen months'* instruction in two collegiate years.

The Course will consist of daily lectures and examinations upon all the departments or branches usually taught here or elsewhere. Chemical instruction will also form an important feature of this Course. The City Dispensory established at the College building, with the number of cases both medical and surgical, which present themselves from the adjacent country, offer sufficient material to occupy one hour each day to this department of medicine.

Those attending this Course will be formed into classes, and in regular order will have an opportunity of examining patients, prescribing, &c., always, however, in the presence and subject to the criticisms of the Professor conducting the Clinic.

This Course will not be recognized as a *regular* Course, nor will it be regarded as a pre-requisite to graduation.

